# Review of Institute of Medicine and National Research Council Recommendations for One Health Initiative

**DOI:** 10.3201/eid1912.121659

**Published:** 2013-12

**Authors:** Carol Rubin, Tanya Myers, William Stokes, Bernadette Dunham, Stic Harris, Beth Lautner, Joseph Annelli

**Affiliations:** Centers for Disease Control and Prevention, Atlanta, Georgia, USA (C. Rubin, T. Myers);; National Institutes of Environmental Health Sciences, Research Triangle Park, North Carolina, USA (W. Stokes);; US Food and Drug Administration, Rockville, Maryland, USA (B. Dunham);; Department of Homeland Security, Washington DC, USA (S. Harris) US Department of Agriculture, Des Moines, Iowa, USA (Lautner);; US Department of Agriculture, Riverdale, Maryland (J. Annelli)

**Keywords:** zoonoses, animal diseases, veterinary medicine, public health, environmental medicine, vectors, disease reservoirs, medicine, world health

## Abstract

Human health is inextricably linked to the health of animals and the viability of ecosystems; this is a concept commonly known as One Health. Over the last 2 decades, the Institute of Medicine (IOM) and the National Research Council (NRC) have published consensus reports and workshop summaries addressing a variety of threats to animal, human, and ecosystem health. We reviewed a selection of these publications and identified recommendations from NRC and IOM/NRC consensus reports and from opinions expressed in workshop summaries that are relevant to implementation of the One Health paradigm shift. We grouped these recommendations and opinions into thematic categories to determine if sufficient attention has been given to various aspects of One Health. We conclude that although One Health themes have been included throughout numerous IOM and NRC publications, identified gaps remain that may warrant targeted studies related to the One Health approach.

Over the past decade, animal and human health leaders have begun to consider the benefit of collaboration, prompted by recognition that highly specialized practices of veterinary and human medicine are missing inextricable links between human health, animal health, and the viability of ecosystems. The 2008 Final Report of the American Veterinary Medical Association (AVMA) One Health Initiative Task Force defined One Health as the collaborative effort of multiple disciplines—working locally, nationally, and globally—to attain optimal health for people, animals and our environment. The report included the recommendation that the AVMA, the American Medical Association, and other interested parties should “plan a study on One Health to be conducted by the National Academy of Sciences and secure the necessary funding to underwrite this effort” (*1*). In 2009, the Institute of Medicine (IOM) and National Research Council (NRC) co-hosted the One Health Commission Summit, described as “a forerunner to an IOM study on One Health…[that will be] used to develop a strategic roadmap for public and private policies and initiatives that will, in turn, be instrumental in shaping the implementation of the One Health vision, both domestically and internationally” (*2*). The study was slated to begin in 2010; however, funding required to initiate it has not yet been committed.

A review of existing IOM publications for One Health–related consensus recommendations or individual opinions is a critical step in assessing whether to move forward with a general, or more refined, focus that will complement the existing body of IOM/NRC reports. We sought to complete such a review, and to fit the findings into a framework that would facilitate a data-driven assessment of how to move forward in possibly seeking an IOM/NRC review of One Health.

## Methods

### The National Academies and Their Reports

A primary function of the National Academies is to provide unbiased and timely expert advice to policy makers and the general public. The National Academies include the National Academy of Sciences, the IOM, the NRC, and the National Academy of Engineering. Their operations are independent from the US federal government and not funded by direct appropriation, although studies are often mandated by Congress in the interest of seeking expert counsel. Studies can be requested by federal agencies or by independent organizations.

At the National Academies there is a vast difference in the weight ascribed to consensus committee recommendations, compared to the individual opinions that are collected in a workshop summary. A consensus committee, typically including 10–15 members, is carefully chosen to represent a range of specific disciplines and experiences. Consensus committees are carefully structured to ensure that all members are independent of the sponsoring agencies (*3**–**5*). The committees operate under a set of rigorous rules pertinent to Section 15 of the Federal Advisory Committee Act. Each committee member undergoes an extensive bias and conflict of interest review, and their names are posted online for public comment. The committee collects information from presentations, literature reviews, and other means; the committee’s recommendations are then designed in a very structured way. When a draft report is compiled, it is submitted to a review committee with a similar mix of disciplinary expertise. The entire process is overseen by the overarching National Academies Report Review Committee (RRC). 

In strict contrast, a workshop summary is not allowed to contain anything that could be interpreted as a consensus conclusion or recommendation. It is not reflective of a Federal Advisory Committee Act process and the RRC is minimally involved in most instances. To separate the workshop summary from the report of the persons who designed the workshop, the summary is always written by an appointed reporter rather than a workshop planning committee member . The goal is to ensure that the workshop report is not seen as the product of a committee process, but as a collection of opinions expressed by workshop participants. The standard of peer review for a workshop is very different from that described above for a consensus study. A much smaller group of reviewers is involved, and the objective of the review is to ensure that the report is an accurate and clear description of what happened, not what should have happened. Because there is some overlap in content between the IOM/NRC consensus reports and IOM workshop summaries in terms of coverage of health-related issues, we included both types of reports in this review.

### Selection of Reports for Review

Titles and objectives of NRC/IOM reports during 1991–2013 were reviewed to identify content addressing interactions among humans, animals, and the environment. By using this process, 20 reports (Table) (*6**–**25*) were judged most likely to contain multiple recommendations or opinions related to One Health concepts. Although it is likely that additional reports may contain One Health concepts, this review was constructed to provide a starting point to inform those considering how future studies of One Health by the National Academies could be constructed.

**Table:** Listing of Institute of Medicine/National Research Council reports included in review, 1991–2013*

Date	Title	Type
**1991**	**Animals as sentinels of environmental health**	**Report**
**1992**	**Emerging infectious diseases: Microbial threats to health in the United States**	**Report**
**1999**	**The use of drugs in food animals: Benefits and risks**	**Report**
2001	Emerging infectious diseases: From the global to the local perspective	WS
2002	The emergence of zoonotic diseases: Understanding the impact on animal and human health	WS
**2003**	**Microbial threats to health: Emergence, detection and response**	**Report**
**2005**	**Animal health at the crossroads: Preventing, detecting, and diagnosing animal diseases**	**Report**
**2005**	**Critical needs for research in veterinary science**	**Report**
2006	Addressing foodborne threats to health: Policies, practices, and global coordination	WS
2006	The impact of globalization on infectious disease emergence and control	WS
2007	Global infectious disease surveillance and detection: Assessing the challenges – finding solutions	WS
2008	Vector-borne diseases: Understanding the environment, human health and ecologic consequences	WS
**2009**	**Sustaining global surveillance and response to emerging zoonotic diseases**	**Report**
2010	Antibiotic resistance: Implications for global health and novel intervention strategies	WS
2010	Infectious disease movement in a borderless world	WS
**2011**	**Climate change, the indoor environment, and health**	**Report**
2011	Critical needs and gaps in understanding prevention, amelioration, and resolution of Lyme and other tick-borne diseases	WS
2011	Fungal diseases: an emerging threat to animal, human and plant health	WS
2011	The causes and impacts of neglected tropical and zoonotic diseases: Opportunities for integrated intervention strategies	WS
2012	Improving food safety through a One Health approach	WS

*IOM, Institute of Medicine; NRC, National Research Council; Report and bold text indicates recommentations from consensus reports, NRC committee reports or IOM consensus report;s WS, IOM workshop summary or workshop report.

### Defining One Health Concepts

For the purposes of this review, a consensus recommendation or workshop opinion was deemed related to One Health concepts if it included any aspect of the relationships between humans, animals, and the ecosystems in which they coexist and interact. Although this definition may be viewed as broad, it was chosen intentionally to prevent bias for or against any particular component of One Health.

### Grouping of Recommendations

All consensus reports and some workshop summaries and workshop reports included summary or overview chapters containing an aggregated view of consensus report recommendations or themes emerging from the workshop. However, in cases in which reports lacked such an organized set of recommendations, the full report was reviewed to determine whether any pertinent information was conveyed within individual chapters. Recommendations or opinions found to be related to One Health were compiled for each individual report; then the aggregated list was reviewed to identify common themes. Finally, we sought to identify examples of completed or ongoing activities that address recommendations and opinions.

## Results

Of the 20 publications that were reviewed in depth for this article, 8 were consensus reports. More than 50 recommendations and opinions were extracted, covering a broad array of topics ranging from a specific disease, system, or policy improvement, to general statements encouraging expansions of partnerships and broad investments in infrastructure for surveillance systems. As expected, the strongest and most specific recommendations were captured in consensus reports.

We grouped the recommendations and opinions into 7 topical categories: Surveillance and Response, Governance and Policy, Laboratory Networks, Training Needs, Research Needs, Communication Needs, and Partnerships. Technical Appendix Table 1 displays a paraphrased listing of the recommendations by category. The list of recommendations was circulated among the authors and other subject matter experts in an attempt to identify ongoing activities or programs that appear to address gaps identified in the IOM and NRC reports. Technical Appendix Table 2 lists all of the recommendations, the exact references that support each recommendation, and examples of activities that appear to respond to specific recommendations.

## Discussion

On the basis of the list compiled from the 20 reviewed reports, we found that the principles of One Health have, to varying extents, been included in many of the NRC/IOM recommendations and IOM workshop summaries. All of the reviewed reports had at least 1 recommendation related to an aspect of One Health. This sample was, admittedly, skewed toward those reports most likely to include recommendations, but we were impressed with the quantity identified in this review. Although even the earliest (1991) consensus report reviewed contained recommendations, a deeper review including reports from earlier dates would likely find additional recommendations linked to One Health concepts. As might be expected, One Health (or a related term) was not used in all instances as a descriptor for recommendations or opinions that fit within the definition of One Health activities used for this review; in fact, many recommendations that by today’s understanding are clearly related to One Health were not tagged as such.

The quantity of recommendations and workshop opinions related to One Health concepts suggests that a reasonable level of attention has been given to the One Health movement in the past 2 decades of IOM/NRC publications. However, level of coverage does not necessarily translate into sufficient consideration of all aspects of a One Health approach, nor does it indicate adequate consideration of current understandings of One Health concepts. Apportioning our findings into thematic categories let us create a framework for evaluation of breadth of coverage. We found that some categories have received more attention than others. For example, the Surveillance and Response category had 16 recommendations or opinions that originated from 14 individual reports; and the Governance and Policy category had 10 recommendations or opinions from 8 reports. By contrast, 4 recommendations or opinions were identified in the Partnership category, and 3 were identified in the category of Communication Needs.

Most of the examples of implementation of One Health concepts that are described in the Technical Appendix are not directly associated with specific IOM or NRC recommendation. In contrast, recommendations from the United States Agency for International Development (USAID)-supported IOM report “Sustaining Global Surveillance and Response to Emerging Zoonotic Diseases” (2009) were translated into One Health activities under USAID’s Emerging Pandemic Threats (EPT) Program. Progress in One Health activities may be a result of explicit recommendations from IOM and NRC reports, or simply be occurring because of increasing awareness of One Health concepts.

### Examples of Progress

In the Surveillance and Response category, there is good evidence that progress has been made in addressing some of the One Health–related recommendations generated from IOM and NRC studies. Several recommendations in this category address the need for integrated surveillance systems that capture information from multiple sectors. An excellent example of such integrated surveillance is the National Antimicrobial Resistance Monitoring System, which became operational in 1996 as a collaborative effort of the Centers for Disease Control and Prevention (CDC), the US Food and Drug Administration, and the US Department of Agriculture. The National Antimicrobial Resistance Monitoring System tracks antimicrobial susceptibility among enteric bacteria from humans, retail meats, and food animals (*26*–*28*) and provides timely integrated surveillance information that has enhanced the effectiveness of response to outbreaks of enteric disease. Although many of the recommendations regarding surveillance and response have been addressed in part, this particular area may provide an opportunity for a more focused IOM study group to evaluate how existing systems could be linked or merged to provide a sustainable, integrated surveillance system that addresses the needs of multiple sectors.

Recommendations in the Governance and Policy category appear not to have been specifically addressed and may represent a gap that needs to receive more attention. However, a One Health website, www.onehealthglobal.net, was released in mid-April 2012. The portal is intended to be a network-of-networks that speaks to One Health governance and that may serve as a mechanism that facilitates the recommendations within the Governance and Policy category (*29*). The portal, Operationalizing “One Health”: A Policy Perspective—Taking Stock and Shaping an Implementation Roadmap (*30*), is a product of the One Health Global Network Work Group that was formed as an outcome of the “Stone Mountain” meeting organized by CDC in collaboration with the World Organisation for Animal Health (OIE), the United Nations Food and Agriculture Organization (FAO), and the World Health Organization (WHO). 

Laboratory network recommendations have been addressed on several national fronts, including planning for a National Bio and Agro-Defense Facility (*31*) and the flourishing National Animal Health Laboratory Network (*32*). Internationally, OIE, FAO, and WHO have received USAID EPT funds to improve networking among human and animal laboratories (*33*). As mentioned previously, this EPT funding occurred after a 2009 Consensus Report, demonstrating direct actions to enhance laboratory capabilities in response to recommendations made within an IOM report (*18*)

Within the Training category, some recommendations are being addressed by the Stone Mountain Meeting Training Workgroup, grantees from 1 of 4 EPT projects named RESPOND, and the University of Minnesota with Rockefeller Foundation funding (*34*). These 3 groups work independently and also collaboratively to define One Health Core Competencies for varying levels of practitioners. They also develop course catalogs that capture existing training opportunities and identify needed training materials. In April 2012, the University of Florida announced that it will offer 2 new One Health degree programs, including a PhD in Public Health with a One Health concentration. “The One Health concentration is a research-oriented health degree that emphasizes working across public health, veterinary health, and environmental health disciplines to tackle difficult health problems” (*35*).

Similar selected examples of programs and projects that address the IOM recommendations can be identified for the categories of Research Needs (e.g., National Institutes of Health [NIH] Centers of Excellence for Influenza Research and Surveillance program, EPT PREDICT projects and the NIH-NSF Ecology and Evolution of Infectious Diseases Program: A Joint Program for Multidisciplinary Research [*36*]), Communication Needs (e.g., formation of One Health Offices at USDA and CDC), and Partnerships (e.g., US Interagency One Health Working Group, inclusion of veterinarians in CDC Field Epidemiology and Laboratory Training Programs). Although these examples are excellent steps in the right direction, they do not respond to the majority of the recommendations. In particular, recommendations that point toward collaboration, resource sharing, coordinated research, and strengthened lines of communication deserve greater attention.

## Conclusions

The quantity of recommendations found suggests that, on a relatively consistent basis, One Health concepts have been considered to be part of working group deliberations, and of IOM and NRC studies, although there is no single entity or process for tracking progress on the recommendations of the National Academies’ studies related to One Health. The examples we provide of completed, ongoing, and planned activities that address the recommendations are not intended to be comprehensive; however, the examples demonstrate that the One Health approach is making inroads. If additional IOM or NRC studies addressing One Health do go forward, we suggest that progress to date be considered and that the questions posed by the National Academies be carefully targeted to address remaining gaps.

Technical Appendix Published recommendations for One Health activities, 1991–2013.
