# Supplementary material for: Review of Institute of Medicine and National Research Council Recommendations for One Health Initiative
Source: Emerg Infect Dis. 2013 Dec;19(12):1913–7. doi: 10.3201/eid1912.121659 (PMC3840875; doi:10.3201/eid1912.121659)
Supplement: Technical Appendix — Published recommendations for One Health activities, 1991–2013. [file 12-1659-Techapp-s1.pdf]

Article DOI: http://dx.doi.org/10.3201/eid1912.131659

Review of Institute of Medicine and National Research Council Recommendations for One Health Initiative

Technical Appendix

Published Recommendations for One Health Activities, 1991–2013.

Technical Appendix Table 1. One Health Recommendations in Institute of Medicine and National Research Council Publications, 1991–2013*

Category	Abbreviated recommendations
Surveillance and Response	**Evaluate adequacy of global wildlife surveillance, inclusion in existing national systems, and sentinel value**
	**Make global surveillance networks sustainable**
	**Collaborate with other countries and international organizations to create global systems for preventing, detecting, and diagnosing diseases as they related to animal and human health**
	**Create independent audit and rating system for national surveillance systems with respect to emerging zoonotic disease outbreaks**
	**Evaluate value of domestic animals as sentinels and in disease surveillance and detection**
	**Implement surveillance on effect of outdoor and indoor conditions as well as building characteristics on occupant health**
	Strengthen links between human, domestic animal, wildlife and vector surveillance systems
	Incorporate allied factors (climate, extreme weather, vegetation, etc.) into global surveillance systems
	Coordinate response from animal and public health authorities to foodborne outbreaks
	Evaluate if and how to incorporate syndromic surveillance
	Create tools for cost-effectiveness analysis of current surveillance systems
	Analyze feasibility and benefits of incentives for reporting to surveillance systems
	Ensure adequate systems for global surveillance and response, including engagement of all stakeholders
	Recognize human travelers as sentinels as well as agents of dispersal in the context of fungal diseases
	Increase capacity for early detection (and response) to fungal "invasions"
	Improved sharing of data internationally so that clusters of food-related enteric disease can be identified
Governance and Policy	**Form expert committee to oversee development/use of antibiotics in human and food-animal medicine**
	**Develop and implement integrated and standardized regulations nationally to address exotic animal trade.**
	**Enhance World Organisation for Animal Health (OIE) authority to achieve compliance in reporting**
	**Establish coordinating body to facilitate development and implementation of integrated surveillance/response to zoonotic diseases (led by USAID and other stakeholders)**
	**Negotiate international agreement on trade in wildlife species**
	Create interdisciplinary animal-public health programs
	Develop effective and comprehensive zoonoses management program with national leadership from the zoonosis community
	Expand U.S. rules to allow testing of animal specimens when needed for human therapy/outcome measures, and implement similar quality requirements as for human samples
	Reform legislation and regulations to address appropriate antiobiotic use and incentivize novel antibiotics
	Focus on wildlife markets to reduce trade that threatens human, animal and ecosystem health
Laboratory Networks	**Expand and strengthen animal health laboratory network to ensure capacity for routine and emergency needs; link all parties (federal, state, university and commercial) involved in animal and zoonotic diagnoses**
	Strengthen veterinary diagnostic laboratories, and fund or reduce lab fees to encourage submissions
	Increase national lab capacity at BSL3/4 and veterinary BSL4
	Strengthen and integrate laboratory networks diagnosing food-borne and animal diseases
Training Needs	**Develop interdisciplinary disease centers to promote multi-disciplinary approaches to microbial threats**
	**Build veterinary capacity to support public health, food systems, biomedical research, diagnostic laboratory investigations, pathology, epidemiology, ecosystem health and food animal practices**
	Develop and strengthen linkages between public health training in medical and veterinary programs

Category	Abbreviated recommendations
	Improve understanding of cultural, infrastructure, and other issues affect improvement of surveillance, control and management of diseases
	Conduct training programs in food safety for public health officials, veterinarians and the animal health community in developing countries
	Support training of medical acarologists and tick biologists to ensure continuing progress on tick-borne diseases
Research Needs	**Expand and coordinate NIH-supported research on agent, host, vector and environment factors leading to emergence of infectious diseases**
	**Increase funding for basic research on antibiotics, including development of more rapid and wide-screen diagnostics to improve tracking of emerging resistance and zoonotic diseases**
	**Climate change research should incorporate health issues associated with indoor environment**
	Fund, sustainably, research in knowledge gaps in ecology, epidemiology, and pathogenesis of zoonotic diseases
	Investigate ecology of foodborne diseases to inform integration of animal and human health surveillance
	Define role of water as source of foodborne illness
	Provide evidence of economic benefit and value to investors to successfully solicit support for additional private or public research funding
	Integrate research efforts and findings on infectious diseases in humans, animals, and plants
	Develop comprehensive national databases that capture ecosystem, vector and patient data related to Lyme disease
	Develop bioeconomic models to assess economic impact of invasive species and evaluate prevention and mitigation
	Develop proof-of-concept prototypes for validation of One Health approach to food safety in developing world and to public-private partnerships
Communication Needs	**Develop trust and communication pathways between industry, public sector, academia, NGOs, smallholder farmers and community representatives to achieve bi-directional flow of formal and informal information needed for evidence-based decision making and coordinated actions**
	Educate public on complexities of antimicrobial resistance, especially related to use in food animals
	Develop public campaigns to address bush meat and exotic animal awareness
Partnerships	**Develop/enhance EPA/CDC efforts to identify and mitigate health risks from degradation in indoor environmental quality associated with climate change**
	Develop tripartite cooperative program within federal agencies to address infectious diseases in humans, domestic animals and wildlife, and to catalyze development of similar programs at state level that would network with the federal cooperative program
	Recognize the primacy of prevention and control of human disease, and improve collaboration between public health and agricultural agencies
	Establish prevention of invasive species spread as international public good and assist developing countries in establishing capacity for surveillance, detection and prevention of biologic (fungal) invasions

*Bolded and shaded rows indicate recommendations from consensus reports

Technical Appendix Table 2. One Health-related recommendations identified in 17 Institute of Medicine or National Academies studies, divided into 7 thematic groups. Each recommendation is referenced to the original report(s) and examples of past, current or planned activities that address the recommendation are provided. A "Three Bears" review of One Health concepts in IOM studies: too much, too little, or just right?

Thematic group	Reference(s)	Examples of related activities
**Surveillance and Response**		
**Evaluate whether global wildlife surveillance is adequate, include wildlife in existing national surveillance with links to agricultural intelligence, and evaluation of wildlife for value as sentinels**	**IOM CR 2009, NRC CR 1991; also discussed in IOM WS 2011 (Fungal Diseases) and IOM WS 2002**	**USAID Emerging Pandemic Threats Program (*1*)**
**Financial sustainability of global surveillance networks should be stabilized**	**IOM CR 2009; also discussed in IOM WS 2002**	
**"The United States should commit resources and develop new shared leadership roles with other countries and international organizations in creating global systems for preventing, detecting, and diagnosing known and emerging diseases, disease agents, and disease threats as they relate to animal and public health"**	**NRC CmR 2005 (Animal Health)**	**USAID Emerging Pandemic Threats Program (**2**)**
**Independent audit and rating is needed of "national surveillance system capacities for detecting and responding to emerging zoonotic disease outbreaks in humans and animals"**	**IOM CR 2009**	
**Evaluate domestic animals for value as sentinels and significance for disease**	**NCR CR 1991; also discussed in IOM WS 2011 (Fungal Diseases), IOM WS**	**World Small Animal Veterinary Association (WSAVA) (**3**)**

Thematic group	Reference(s)	Examples of related activities
**surveillance/detection**	**1992**	
**"The Environmental Protection Agency and other federal agencies should put into place a public-health surveillance system that uses existing environment and health survey instruments to gather information on how outdoor conditions, building characteristics, and indoor environmental conditions are affecting occupant health and on how these change over time."**	**IOM CR 2011**	
Strengthening links in human and animal surveillance: "Strengthening disease surveillance in humans and domestic animals. Priority areas include improving communication and information sharing between the medical and veterinary communities and designing integrated medical and veterinary disease surveillance systems at a regional level."[2001] and "…collaboration among practitioners of veterinary and human medicine, along with overlapping surveillance systems, would be highly beneficial to both people and animals"[2011] "Because many TBDs [tick borne diseases] are zoonotic, animal and human health experts urgently need to collaborate and to develop an integrated surveillance system that includes domestic animals, wildlife, ticks and people. Wider and more effective surveillance could allow animals to serve as sentinels and surrogates for human risk and exposure to TBDs."[2011]	IOM WS 2011 (Lyme and other TBD), IOM WS 2001	National Antimicrobial Monitoring System (NARMS):<br>CDC (4)<br>FDA (5)<br>USDA 6)<br>Global Foodborne Infections Network (7)<br>Training materials developed by FDA/CDC/USDA to assess community readiness for food emergencies (8)<br>ArboNET: National surveillance system for arboviral diseases in the United States (*9*)<br>National Biosurveillance Integration Center (NBIC) (*10)*
Determine how to incorporate climate, extreme weather events, vegetation, insect vector distribution, etc. into global surveillance systems	IOM WS 2011 (Fungal Diseases), IOM WS 2008, IOM WS 2007, IOM WS 2002	National Ecologic Observatory Network (NEON) (*11*)
Coordination of responses to foodborne outbreaks from animal and public health authorities	IOM WS 2006 (Foodborne)	FREE-B Tool for Food-Emergency Readiness<br>(*12*)<br>Interagency Foodborne Outbreak Response Working Group (*13*)<br>FDA's CORE Network: Coordinated Outbreak Response & Evaluation<br>(*14*)
Evaluate if/how to incorporate syndromic surveillance	IOM WS 2007	Pilot study to evaluate feasibility of syndromic surveillance for Ontario swine industry (*15*)
Tools for cost effectiveness analysis of current surveillance systems	IOM WS 2007	
Analysis of feasibility/benefit of incentives for reporting to surveillance systems	IOM WS 2007	
Advancement of OH will require "adequate systems and capacities to conduct global surveillance and respond to public health emergencies" and engagement "of all stakeholders, and particularly the private sector in global disease surveillance and response, recognizing that some key groups do not perceive such action to be in their best interest"	IOM WS 2010 (Infectious Disease Movement)	Incorporation of animal and human health in country-specific influenza response plans such as the 2012 North American Plan for Animal and Pandemic Influenza (NAPAPI) retains the key elements of the 2007 version, while incorporating the lessons learned from the North American response to Pandemic (H1N1) 2009, including recognizing that a pandemic influenza virus may emerge in our region and expanding the focus on animal influenza viruses to incorporate both avian and non-avian species. (*16*)
"Recognizing the importance of human travelers as disease couriers, transmitters,	IOM WS 2011 (Fungal Diseases)	

Thematic group	Reference(s)	Examples of related activities
and sentinels and, therefore, a critical target for infectious disease surveillance and detection”		
“Increasing capacity for the early detection of, and rapid response to, biological invasions”	IOM WS 2011 (Fungal Diseases)	Global Early Warning and Detection System (GLEWS) a joint system that builds on the added value of combining and coordinating the alert and disease intelligence mechanisms of OIE, FAO and WHO for the international community and stakeholders to assist in prediction, prevention and control of animal disease threats, including zoonoses, through sharing of information, epidemiologic analysis and joint risk assessment. (*17*)
Increased international exchange of molecular and epidemiologic data “to enable the sequence based linking of clusters of viral enteric disease, and thereby to track global food-borne outbreaks—outbreaks that threaten to produce more virulent viruses through recombination”	IOM WS 2012	
**Governance and Policy**		
**“The committee recommends that further development and use of antibiotics in both human medicine and food-animal practices have oversight by an interdisciplinary panel of experts composed of representatives of the veterinary and animal health industry, the human medicine community, consumer advocacy, the animal production industry, research, epidemiology, and the regulatory agencies.”**	**NRC CmR 1999**	**A Public Health Action Plan to Combat Anitmicrobial Resistance (*18*). Interagency Task Force participants include CDC, FDA, NIH, AHRQ, CMS, HRSA, USDA, DOD, VA and EPA.**
**“Integrated and standardized regulations should be developed and implemented nationally to address the import, sale, movement, and health of exotic, non-domesticated and wild-caught animals.”**	**NRC CmR 2005 (Animal Health)**	
**Enhance OIE authority to achieve compliance with existing reporting obligations**	**IOM CR 2009**	
**“USAID, in cooperation with the UN and other stakeholders from human and animal health sectors, should promote the establishment of a coordinating body to ensure progress toward development and implementation of harmonized, long-term strategies for integrated surveillance and response for zoonotic diseases.”**	**IOM CR 2009**	
**Review of current trade/import situations to quantify risk of bush meat import and wildlife trade (both legal and illicit), to be followed by negotiations on “a new international agreement on trade in wildlife species that improves international collaboration on reducing the threat that such trade presents to human and animal health.”**	**IOM CR 2009, IOM WS 2012**	**The Economics of Agricultural and Wildlife Smuggling, USDA, Economics Research Services Report Number 81, September 2009. (*19*)**
“Create interdisciplinary animal-public health programs.”	IOM WS 2006 (Foodborne)	A Federal Interagency One Health Working Group was established in 2010 and expanded in 2012 with the intent of furthering such an interdisciplinary approach to animal-public health (and environmental health) programs.
National leadership from the zoonosis community behind an effective/comprehensive program to manage	IOM WS 2002	Coordinating Zoonotic Disease Surveillance: Partnering Agriculture and Public Health, NAHSS Outlook June

Thematic group	Reference(s)	Examples of related activities
zoonoses (with CDC's emerging disease plan as a possible model)		2005 (*20*)
Expanded (U.S.) government rules to allow for testing of animal specimens when results may affect human therapy/outcome, and requirements that labs performing such testing participate in proficiency testing/QA programs (CLIA) [note: rabies given as an example here, not certain of need for other diseases]	IOM WS 2002	
Policy makers should develop and support legislation/regulatory reforms to address appropriate use of antibiotics and incentivize development of novel antibiotics	IOM WS 2010 (Antibiotic Resistance)	
Focusing efforts on markets (e.g., wildlife markets) to regulate, reduce, or eliminate trade that threatens the health of humans, domestic animals, wildlife, and ecosystems.	IOM WS 2011 (Fungal Diseases)	
**Laboratory Networks**		
**"The animal health laboratory network should be expanded and strengthened to ensure sufficient capability and capacity for both routine and emergency diagnostic needs and to ensure a robust linkage of all components (federal, state, university, and commercial laboratories) involved in the diagnosis of animal and zoonotic diseases."**	**NRC CmR 2005 (Animal Health)**	**Integrated Consortium of Laboratory Networks (ICLN) – an operational system of laboratory networks coordinated by DHS for early detection and management of events requiring an integrated laboratory response (*21*)**<br>**National Animal Health Laboratory Network (NAHLN) (*22*)**<br>**OIE/FAO Network of Expertise on Animal Influenza – established jointly by OIE and FAO to support and coordinate global efforts to prevent, detect and control critical influenzas in animals (*23*)**<br>**Swine Influenza Surveillance in the U.S. (*24*)**<br>**CDC's Laboratory Response Network – includes both public health and veterinary laboratories (*25*)**<br>**FDA/USDA Food Emergency Response Network (*26*)**
Strengthen veterinary diagnostic laboratories, and encourage submissions by funding/reducing lab fees	IOM WS 2002	National Animal Health Laboratory Network (NAHLN) (*27*)
Increased number/capacity of national laboratories with BSL3/4 and veterinary BSL4 capabilities, able to run multi-pronged research programs and scalable on outbreaks	IOM WS 2002	National Bio and Agro-Defense Facility (*28*)<br>NIH National and Regional Biocontainment Laboratories (*29*)
"Strengthen and integrate laboratory networks that diagnose food-borne and animal diseases.	IOM WS 2006 (Foodborne)	
**Training Needs**		
**"Interdisciplinary infectious disease centers should be developed to promote a multidisciplinary approach to addressing microbial threats to health."**	**IOM CR 2003**	
**"Industry, producers, the American Veterinary Medical Assocation (AVMA), government agencies, and colleges of veterinary medicine should build veterinary capacity through both recruitment and preparation of additional veterinary graduates into careers in public health, food systems, biomedical research, diagnostic laboratory investigation, pathology, epidemiology, ecosystem health, and food animal practice."**	**NRC CmR 2005 (Animal Health)**	

Thematic group	Reference(s)	Examples of related activities
Linkages between and public health training in medical and veterinary programs	IOM WS 2006 (Globalization), IOM WS 2012	The Stone Mountain Meeting Training Working Group found 34 University efforts in One Health designed to establish formal linkages between medical and veterinary education. Below are just a few examples:<br>-Triangle Global Health Consortium (*30*)<br>-University of Wisconsin-Madison (*31*)<br>-University of Illinois at Urbana-Champaign (*32*)<br>-Yale School of Medicine (*33*)<br>-University of Florida (*34*)<br>-UC Davis School of Veterinary Medicine (*35*)
"…critical to improve our general understanding of the various cultural, infrastructure, and other issues that will affect how the United States can best work with the world community to improve the surveillance, control, and management of disease."	IOM WS 2002	
"Conduct training programs in food safety for public health officials in developing countries, veterinarians, and the animal health community."	IOM WS 2006 (Foodborne), IOM WS 2012	
"The number and training of medical acarologists and tick biologists are declining, and scientists who do investigate TBDs often focus only on Lyme disease-related questions. Support for the training of tick biologists with wide-ranging interests and broad research portfolios are essential to ensure continued progress on the full spectrum of TBDs."	IOM WS 2011	
**Research Needs**		
**"The committee recommends the expansion and coordination of National Institutes of Health-supported research on the agent, host, vector, and environmental factors that lead to emergence of infectious diseases. Such research should include studies on the agents and their biology, pathogenesis, and evolution; vectors and their control; vaccines; and antimicrobial drugs."**	**IOM CR 1992**	**NIEHS-NIAID Workshop to examine the interactions between environmental exposures and infectious agents in the etiology of human diseases. Sept 8–9, 2011 (*36*)**<br>**Centers for Oceans and Human Health Research Program, NIEHS and NSF (*37*)**<br>**NIH-NSF Ecology and Evolution of Infectious Diseases Program: A Joint Program for Multidisciplinary Research (2011) (*38*)**
**"The committee recommends increased funding for basic research that explores and discovers new or novel antibiotics and mechanisms of their action, including the development of more rapid and wide-screen diagnostics to improve the tracking of emerging antibiotic resistance and zoonotic disease."**	**NRC CmR 1999**	
**EPA "should spearhead an effort…to make indoor environment and health issues an integral consideration in climate change research and action plans and, more broadly, to coordinate work on the indoor environment and health."**	**IOM CR 2011**	
Knowledge gaps in ecology, epidemiology, and pathogenesis of zoonotic diseases (including vector borne) due to lack of sustained funding	IOM WS 2011 (Lyme and other TBD), IOM WS 2008, IOM WS 2002	NIH-NSF Ecology and Evolution of Infectious Diseases Program: A Joint Program for Multidisciplinary Research (2011) (*38*)
"Examine the ecology of foodborne diseases to inform the integration of animal and health surveillance"	IOM WS 2006 (Foodborne)	

Thematic group	Reference(s)	Examples of related activities
“Define the role of water as a source of foodborne illness”	IOM WS 2006 (Foodborne)	
To successfully solicit support for additional research funding from private and public partners, “researchers will need to provide evidence of economic benefit and opportunities for strategic investment.”	IOM WS 2008	
“Integration of research efforts and findings on infectious diseases in humans, livestock, and wild animals, as well as in crop and wild plants”	IOM WS 2008	
“Informatics to create national databases that capture every aspect of the disease [Lyme] in the ecosystem, the vectors and the patients”	IOM WS 2011 (Lyme and other TBD)	
“Developing bioeconomic models to assess the economic impact of the introduction of invasive species and of alternatives for their prevention and mitigation”	IOM WS 2011 (Fungal Diseases)	
Design “research prototypes for proof-of-concept validation of One Health principles as applied to food safety in the developing world, and also to public-private partnerships between government and the food industry”	IOM WS 2012	
**Communication Needs**		
**“In its work on zoonotic disease surveillance and response, USAID – in collaboration with WHO, FAO, and OIE – should convene representatives from industry, the public sector, academia, nongovernmental organizations (NGOs), as well as smallholder farmers and community representatives to determine how best to build trust and communication pathways among these communities in order to achieve the efficient bi-directional flow of both formal and informal information needed to support effective, evidence-based decision making and coordinated actions.”**	**IOM CR 2009**	
Need to educate public on complexities of antimicrobial resistance, especially given “polarized environment surrounding the issue of antimicrobial use in food animals”	IOM WS 2010 (Antibiotic Resistance)	
Public campaigns to address awareness of risks associated with bushmeat practices and with exotic animal importation	IOM WS 2010 (Infectious Disease Movement)	
**Partnerships**		
**The EPA should collaborate with CDC “to assist state, territorial, and local health and emergency-management agencies in efforts…to identify populations at risk for health problems resulting from alterations in indoor environmental quality induced by climate change and to implement measures to prevent or lessen the problems.”**	**IOM CR 2011**	
“Federal agencies should develop a tripartite cooperative program to address infectious diseases in humans, in domestic animals, and in wildlife. This program should serve as a focus for regular communications through working groups to address information transfer; to improve response to disease emergencies; to establish priorities for collaborative, focused investigations; and to pursue other areas of mutual interest. The program also should serve as a model and catalyst to stimulate the development of	IOM WS 2002	NIH-NSF Ecology and Evolution of Infectious Diseases Program: A Joint Program for Multidisciplinary Research (2011) (38).<br>A Federal Interagency One Health Working Group was established in 2010 and expanded in 2012 with the intent of furthering such an interdisciplinary approach to animal-public health (and environmental health) programs.

Thematic group	Reference(s)	Examples of related activities
similar cooperative programs between state agencies that would network with the federal program."		
"Collaboration can be improved at the internal level as well. Although many international activities have succeeded, often via WHO, difficult circumstances have required the involvement of institutions outside the usual public health agency loop, such as agricultural agencies. This was true when the West Nile virus emerged in the United States in 1999, when the H5N1 influenza virus emerged in Hong Kong in 1997, and when the Hendra virus emerged in Australia in 1994. In each case, turf issues arose, and in some instances efforts to protect agricultural markets seemed to be deemed more important than efforts to protect the public health… The next step in solving such turf issues will involve recognizing the primacy of prevention and control of human disease."	IOM WS 2002	Dedicated One Health Offices at CDC and USDA (39). Multi-Agency Federal Inter-Agency One Health Working Group The national One Health Commission (*40*)
"Establishing the prevention of the spread of invasive species as an international public good, which requires coordination among nation states... Because such a system is only as strong as the "weakest link," efforts are also needed to assist developing nations in establishing capacity for surveillance, detection, and prevention of biologic invasions"	IOM WS 2011 (Fungal Diseases)	
*Bolded and shaded rows indicate recommendations from consensus reports;; IOM, Institute of Medicine; NRC, National Research Council; WS, workshop summary; WR, workshop report; CR, IOM consensus report; CmR = NRC committee report.		

References

1. www.usaid.gov/our_work/global_health/home/News

2. www.usaid.gov/our_work/global_health/home/News

3. http://www.wsava2013.org/

4. http://www.cdc.gov/narms/about_narms.htm

5. http://www.fda.gov/AnimalVeterinary/SafetyHealth/AntimicrobialResistance/NationalAntimicrobialResistanceMonitoringSystem/ucm059089.htm

6. http://ars.usda.gov/Main/docs.htm?docid=6750

7. http://www.who.int/gfn/en/

8. http://www.fda.gov/Food/FoodDefense

9. http://www.iom.edu/~/media/Files/Activity%20Files/Global/ZoonoticDisease/Presentation4Fischer.pdf

10. http://www.dhs.gov/xabout/structure/oha-national-biosurveillance-integration-center.shtm

11. http://neoninc.org/

12. http://www.foodpoisonjournal.com/food-poisoning-resources/fda-announces-free-b-a-new-tool-for-food-emergency-readiness/

13. http://www.fsis.usda.gov/oa/speeches/2000/cw_apfs.htm

14. http://www.fda.gov/Food/FoodSafety/CORENetwork/default.htm?utm_campaign=Google2&utm_source=fdaSearch&utm_medium=website&utm_term=CORE&utm_content=1

15. del Rocio Amezcua M, Pearl DL, Friendship RM, McNab WB. Evaluation of a veterinary-based syndromic surveillance system implemented for swine.. Can J Vet Res. 2010; 74: 241–51.

16. http://www.phe.gov/Preparedness/international/Documents/napapi.pdf

17. http://www.glews.net/

18. http://www.cdc.gov/drugresistance/actionplan/actionPlan.html

19. http://www.ers.usda.gov/Publications/ERR81/ERR81.pdf

20. http://www.aphis.usda.gov/vs/ceah/ncahs/nsu/outlook/issue6/zoonotic_disease_surveillance.pdf

21. https://www.icln.org/

22. http://www.aphis.usda.gov/animal_health/nahln/

23. http://www.offlu.net

24. http://www.aphis.usda.gov/animal_health/animal_dis_spec/swine/siv_surv_manual.shtml

25. http://www.bt.cdc.gov/lrn

26. http://www.fernlab.org

27. http://nahln.org/default/

28. http://www.dhs.gov/files/labs/editorial_0762.shtm

29. http://www.niaid.nih.gov/LabsAndResources/resources/dmid/NBL_RBL/Pages/site.aspx

30. http://triangleglobalhealth.org

31. http://centerforglobalhealth.wisc.edu/173.htm

32. http://vetmed.illinois.edu/

33. http://www.humananimalmedicine.org/

34. http://egh.phhp.ufl.edu/academic-programs/

35. http://www.vetmed.ucdavis.edu/ohi/ceoh/index.cfm#http://www.vetmed.ucdavis.edu/ohi/local-assets/pdfs/loi.pdf

36. http://www.niehs.nih.gov/news/newsletter/2011/october/science-collaboration/index.cfm

37. http://www.niehs.nih.gov/research/supported/centers/oceans/index.cfm

38. http://grants.nih.gov/grants/guide/notice-files/NOT-TW-12-001.html

39. http://www.cdc.gov/onehealth

40. http://www.onehealthcommission.org/
